# Target definition for shipwreck hunting

**DOI:** 10.3389/fpsyg.2015.01615

**Published:** 2015-10-28

**Authors:** Kim Kirsner

**Affiliations:** School of Medicine, University of Notre DameFremantle, WA, Australia

**Keywords:** shipwreck hunting, error, memory, decision making, cognition, mental models, trading zones

## Abstract

The research described in the present article was implemented to define the locations of two World War II shipwrecks, the German raider *Kormoran*, and the Australian light cruiser HMAS *Sydney*. The paper describes the long and complex trail that led through inefficient oceanographic prediction to ambiguous historical prediction involving a single report and on to precise cognitive prediction based on nine reports from more than 70 survivors, a process that yielded a single target position or “mean” just 2.7 NM (nautical miles) from the wreck of *Kormoran*. Prediction for the position of the wreck of *Sydney* opened with wishful thinking that she had somehow reached the coast more than 100 NM away when cognitive analysis of the survivor's reports actually provided the basis for accurate prediction in a position near to the wreck of *Kormoran*. In the account provided below, the focus on cognitive procedures emerged from, first, a review of a sample of the shipwreck hunts, and, second, growing awareness of the extraordinarily rich database available for this search, and the extent to which it was open to cognitive analysis. This review touches on both the trans-disciplinary and the cognitive or intra-disciplinary issues that so challenged the political entities responsible for supervising of the search for the wrecks of *Kormoran* and *Sydney*. One of the theoretical questions that emerged from these debate concerns the model of expertise advanced by Collins (2013). The decomposability of alleged forms of expertise is revealed as a fundamental problem for research projects that might or might not benefit from trans-disciplinary research. Where expertise can be decomposed for operational purposes, the traditional dividing lines between experts and novices, and fools for that matter, are much harder to discern, and require advanced and scientifically informed review.

## Context

HMAS *Sydney* and HSK *Kormoran* sank within an hour or two of each other and approximately 13 nm apart on November 19th, 1941. The British light cruiser and the German raider met by chance while *Sydney* was steaming south from Sunda Strait to Fremantle and *Kormoran* was searching for merchant targets before laying mines off the small coastal port of Carnarvon. The vessels met on a clear afternoon and sighted each other at a distance of 20 or more nautical miles (NM). *Kormoran* turned to the west to avoid combat but *Sydney* followed, and, when *Sydney* closed to less than one NM, combat was inevitable. *Sydney* had squandered her advantages in regard to long range gunnery, director control, armor, and speed. *Kormoran* fired first and the engagement lasted less than 30 nm. *Sydney* was hit by at least fifty 155 mm rounds, hundreds of smaller missiles, and one torpedo, and she sank with the loss of all hands about 5 h later. Kormoran was hit by only three or four six inch rounds but one of those destroyed her motive power and she was scuttled about 6 h after the battle following an orderly disembarkation of the majority of her crew in five lifeboats and two life-rafts. A brief history of the event was published by Gill (1957, 1985).

## Performance criteria

Before the detailed analyses are considered, it is appropriate to identify critical performance criteria for the domain, and to underline the relationship between the performance criteria and the author's focus on the mean. The author has adopted three performance criteria, and one simplifying convention.

The first and most important criterion corresponds to the aim of this edition of *Frontiers*, and the focus on the challenge of identifying an optimal search target or *mean* given six or more forms of evidence, the impact of time on the accuracy of each of those forms, and the inevitable presence of human error. The convention involves the use of “Distance from the wreck of *Kormoran*,” or *Error*, to minimize reliance on the two-dimensional world of traditional cartography. Distances are specified in nautical miles (NM), where one NM = 1.85 km or 1.15 statute miles. The primary challenge for wreck-hunters involved extraction of a mean target position from the reports available for a particular wreck. The first criterion therefore involved *Accuracy*.

The second criterion involved selection of an *efficient* search box, a box that must therefore include the wreck of *Kormoran* while minimizing the size of the search area. The search areas associated with the historical shipwreck searches of interest ranged from 100 Square Nautical Miles or SNM to 600 SNM, however the areas originally tabled for the search for *Kormoran* involved far larger areas than that, up to 13,000 SNM or more in some cases.

The third criterion involved the extent to which a particular solution reflected the power and variety of the available evidence. All other things being equal, a recommendation that reflected one report and one report only must be set aside in favor of a recommendation that reflected several reports or even a substantial fraction of the available evidence. This approach highlighted the weaknesses associated with cherry-picking. For convenience, this criterion is referred to as *Explanatory Power*.

## Disciplines and expertise

The question under review in this paper concerns the location of the wrecks of *Kormoran* and *Sydney*. In retrospect, and with the benefit of hindsight, it is now evident that many of the critical entities in the search were overwhelmed by the shear variety and the depth of the evidence available. The critical issue bears some comparison with the signal detection challenge described by Tanner and Swets (1954) more than half a century ago. But there is another problem. Although Thagard (2005) described the boundary regions between disciplines as a critical venue for innovation in science, the absence of informed scientific leadership among the entities responsible for *management* of the search created an unsympathetic environment for science in general and scientific innovation in particular.

**Figure 1:**
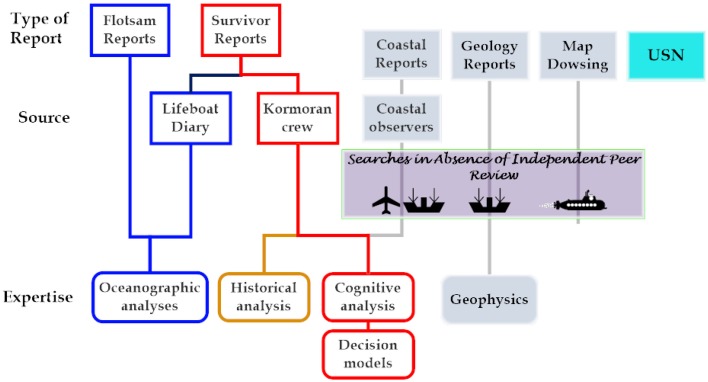
**Overview of the disciplines that contributed to the Search Definition debate**. The figure indicates the source of each type of report, and the form or forms of expertise relevant to the report types, where known. The blue, brown, and red connections reflect the complexity of the relationships among the reports and the disciplines. The gray boxes identify unproductive streams of argument. The aviation and maritime symbols reflect the focus of the WAMM and/or the services in regard to research and search activities.

Figure 1 identifies seven data types and four or possibly five disciplines with an interest in the search for the wreck of Kormoran. The presence of so many interested disciplines reflected the shear variety and the volume of the known and potential sources of data available for the search. The following is a short summary of the available types of evidence and sources:

1. *Flotsam (Oceanography)*: The first type of evidence involved the positions of flotsam, information open to hindcasting to reconstruct the point or points of entry into the water. However, oceanographic hindcasting depends critically on an understanding of the direction, velocity and stability of wind and water currents, and the increasing challenge faced by hindcasting with the passage of time, where time for this search ranged from 84 to 209 h.
2. *Lifeboat diary (Oceanography and Navigation)*: One person in one of the lifeboats maintained a simple diary recording performance data, evidence that enabled reconstruction of the position of *Kormoran*. In practice, interpretation of the diary depended on *oceanographic* as well as *navigational* assumptions, and, if the former are misunderstood, *navigational* reconstruction can be far off the mark.
3. *Reports from Kormoran survivors (History and the Cognitive Sciences):* It is now apparent that the *Kormoran* survivors provided more than 70 reports about the absolute or relative position of *Kormoran*. In addition, RN and RAN servicemen provided nearly 50 summary reports that included comment about the location of the wreck.
4. *Reports from observers on the coast (History and the Cognitive Sciences):* Commencing with journalist Bryan Clark in the 1980's, more than 90 reports were accumulated from about 30 people living on the coast between Geraldton and Dirk Hartog Island.
5. *Magnetic Anomaly (Geophysics*): The first WAMM/RAN search in 1984 was driven by the presence of an anomaly off the coast near Kalbarri, about 130 nm from the wrecks, and received no support from any other source.
6. *Map Dowsing:* Commencing in 1989 Lindsay Knight and Warren Whittaker claimed that a combination of hand-based and electronic-based map dowsing procedures had located the wrecks of *Kormoran* and *Sydney* near the Abrolhos Islands, 180 nm from the position of the wrecks.
7. *The United States Navy (USN)*: Mike McCarthy, Curator of the West Australian Maritime Museum (WAMM), sought assistance from the USN subsequent to the 1991 Oceanography Workshop. The following quotations are from a FAX from the Curator to David Gallo of the Woods Hole Oceanographic Institute (WHOI) in Falmouth, Massachusetts in 1992:
  “My hopes for the search now lie in anti-submarine warfare records, for it has long been my understanding that many of the magnetic anomalies on the seafloor throughout the world are known and have been mapped for strategic purposes. These suspicions have been long since confirmed in discussions with the US, GB, and Australian anti-submarine operatives and were first mooted here in the searches for the SS Koombana many years ago.”

And significantly,

“If the approximate locations of the Sydney/Kormoran are to be found by that route, my problem will be how to keep confidential my source and yet not pretend that we had found the wrecks purely by our own means.”

The overview of the disciplines involved in the search acknowledged the extra-ordinarily rich mixture of evidence, expertise, wishful-thinking, and fantasy that dominated the first 25 years of interest in the search for *Kormoran* and *Sydney* as well as the challenge faced by the private and government entities that engaged in supervision of the search, a challenge they accepted without deploying, seeking, or recognizing the need for expertise.

## Oceanographic and navigation analyses

In 1991 the author approached the WAMM, and proposed that it design and establish an oceanography workshop, the objective of which was to adjudicate between the positions advanced by Montgomery and Barbara Winter, the trigger for the author's initial interest in the project. The first question therefore involved the power of the oceanographic procedures. Could they be used to adjudicate between the positions advanced by Montgomery and Winter?

The rationale for the position advanced by Winter was clear. Winter (1991) included translations of critical elements from Detmers' *Battle Summary*, and the entry for 1700 h on November 19, 1941 included the following, “Straat Malakka 111E 26S.” Winter had tabled the same general position in 1984 on the map shown at page 160. The position was supported in the earlier publication by reference to the statement by Winter that,

“Calculations, ignoring some minor variables, show that the end of nautical twilight on 19th November 1941, latitude 26°S longitude 111°E, was 1901G; the time quoted by Detmers, give or take a minute.”

The critical issue, as recognized by Winter, involved the distinction between the “private” and partially encoded values in the *Battle Summary*, and the “public” positions provided to the RAN interrogators during the Search and Rescue (SAR) and interrogation processes during and following the SAR operation in 1941. The weakness associated with this report involved the probability that the report was intended to be accurate to only the nearest degree, that is 26°S 111°E, as distinct from the nearest minute, that is 26°00′S 111°00′E. Technically, the former involves an area of approximately 3400 Square Nautical Miles (SNM). Justification for the position advanced by Montgomery (1981) was less clear, and relied on selection of one German report, and a dubious claim about the location of the direct route from Sunda Strait to Fremantle.

The original SAR operation conducted by the Royal Australian Air Force (RAAF) and the Royal Australian Navy (RAN) between November 24th and November 29th 1941 yielded eight reports about the locations of flotsam together with 11 reports about the locations of five lifeboats. Two further reports involved the locations of two life-rafts, but these involved chance meetings with passing vessels. The aerial arm of the operation involved approximately nine systematic searches by Hudson aircraft, searches that were dispersed over an area in excess of 30,000 SNM, but searches that were probably too high to detect anything smaller than a lifeboat. In addition, long-range aircraft examined specific targets out to sea, and smaller aircraft searched along the coast. The maritime arm of the search involved some nine ships, and focused on the area where the flotsam was observed.

### Drifting objects

Oceanographic reconstruction could be based on some or all of the known positions of the objects discovered after the battle. The objects comprised two life-rafts, three lifebelts, one float, one dog kennel, and one raft, and they were discovered between 84 and 209 h after the battle. The critical questions therefore concerned the elapsed time for each object, the drifting and/or sailing characteristics of that object, and the direction, velocity and, critically, the variability, of the currents and winds for the period. In addition, as each object had individual characteristics, the analysis had to be applied to each object as an independent entity.

**Figure 2:**
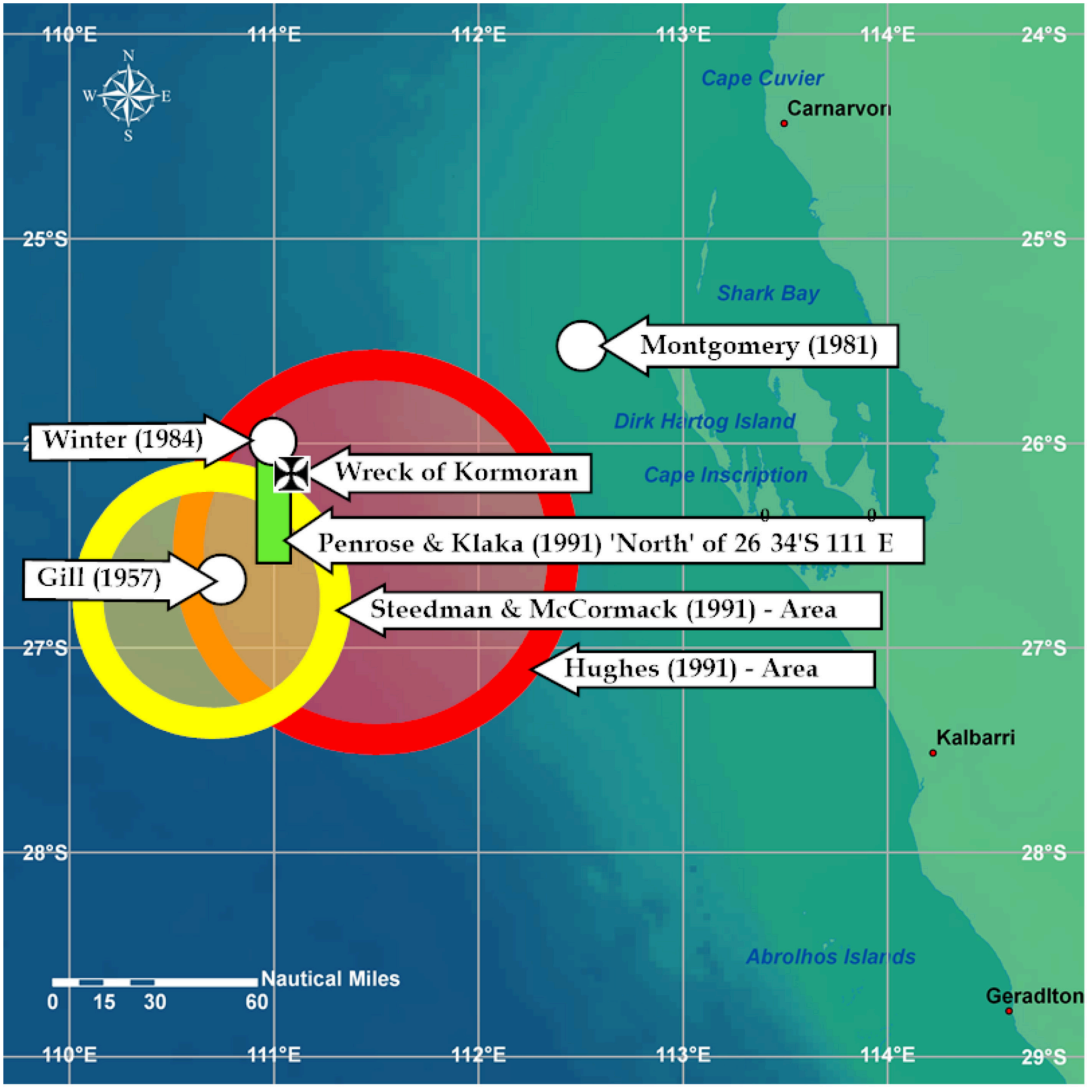
**Depicts positions advanced by Montgomery and Winter and results of 1991 Oceanography Workshop**. The figure includes the recommendation tabled by Gill (1957, 1985) as well as the now known position of Kormoran.

The professional contributions to the 1991 Oceanographic Workshop used hind-casting based on the movements of some or all of the objects that left *Kormoran* or possibly *Sydney* between 1800 and approximately 2300 h on November 19th, 1941. The objects were discovered approximately 120 NM north of the now known position of the wreck of *Kormoran*. The hindcasting analyses typically relied on velocity and bearing information for four variables; current, wind, wind-driven current, and leeway. The workshop yielded four professional reports. The report implemented by Search and Rescue expert Hughes (1991) actually included the position of the wreck of *Kormoran* but the center of the search area was 33 NM from that wreck, and the overall area was ~ 7850 SNM. A second, by oceanographers Steedman and McCormack (1991), involved an area of ~ 1000 SNM, but it did not quite include the wreck of *Kormoran*. A third, by Penrose and Klaka (1991), did not include a search area but it did specify a 30 NM long contour that passed within ~4 NM of the wreck. The fourth analysis, by CSIRO expert Alan Pearce (1991) asserted that the amount of variability in the current and wind values for the area precluded accurate prediction. The first three reports are reflected in Figure 2.

**Figure 3:**
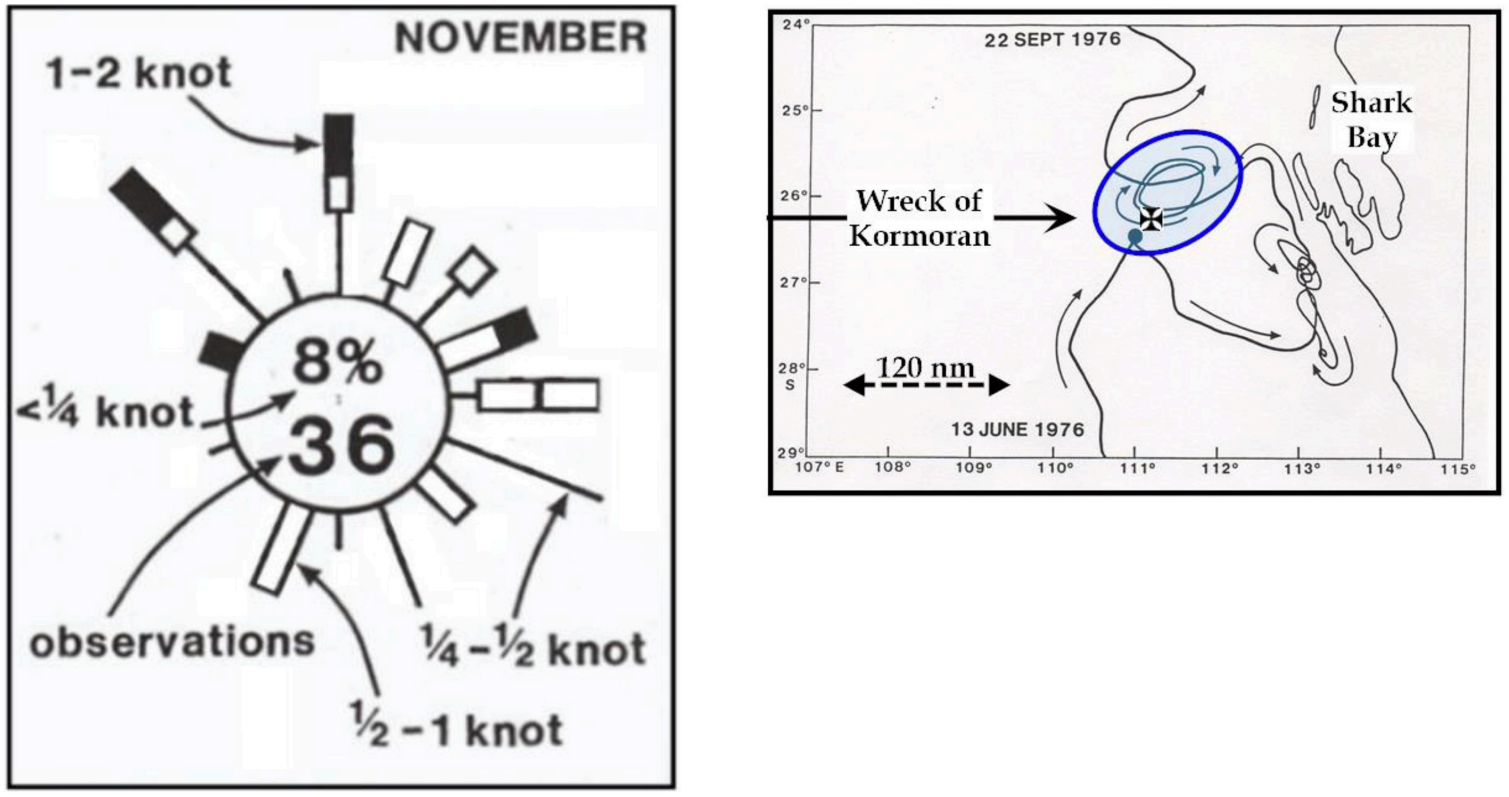
**(A)** Monthly “current rose” from the KNMI Atlas for area encompassed by 25°S, 28°S, 110°E, and 113°E for November. **(B)** Example of oceanic high showing the scale and the type of movement that complicated prediction off the coast of Western Australia.

The challenge posed by Pearce was evident in the current rose re-published by him from the from the KNMI (Dutch) Marine Atlas (See Figure 3A). What the figure highlights is the extraordinary variability in the bearing and velocity of the currents for the area. The figure should be considered in the context of Figure 3B where the presence of huge eddies is noted. The eddies are up to 50 KM in diameter, move in either a clockwise or anti-clockwise direction (for highs and lows, respectively), and the entire system moves gradually from West to East. Furthermore, because the current observations involve almost every point on the compass, the *net drift vector*, the distance made good in any one direction, is very small. Pearce wrapped up his argument in the following terms,

“It is concluded that “climatological” current data cannot be used with any confidence to predict the likely currents which may have carried debris from the HMAS Sydney away from the site of the engagement.”

The role of oceanography for Search Definition was set aside by the author in 1993 for four reasons; first, the size of the error circle defined by Hughes and others was prohibitive for in-water search purposes; second, the argument advanced against reliance on oceanography by CSIRO-expert Alan Pearce underlined doubts about the relevance of the discipline to the search; third, a review that assigned little or no responsibility to oceanography for historically significant searches by Ballard; and, fourth, evolving awareness that the scale and reliability of the reports provided by the survivors might not provide a platform for an efficient search.

### The navigation argument

The critical note for reconstruction of the lifeboat voyage from disembarkation to the coast is attributed to von Malapert, a member of the crew on the lifeboat captained by Henry Meyer, the navigator. The critical extract from Von Malapert's Diary is as follows:

(a) ET0-ET71: 12 h drifting, 59 h sailing at an estimated speed of 1.1 knots 062°, Distance sailed 63 miles; (b) ET71-ET90: Drifted in Force 6–7 winds; (c) ET90-ET134; Sailed for 42 h at an estimated speed of 1.9 knot; Estimated distance = 81 miles.

The Navigation argument can be thought of as one facet of the oceanography analysis. Lifeboats, whether under sail or not, are influenced by the direction, velocity, and duration of the prevailing winds and currents. Steedman and McCormack (1991), a professional oceanographer, reviewed, and rejected analysis of the lifeboat journey, arguing that there were too many unknowns about the sailing and drifting characteristics of the lifeboats to accept the diary for formal analysis (Steedman and McCormack, 1991).

In 2000, shortly after the death of Lindsay Knight, owner/developer of the so-called Knight Direct Location System, a map dowser's dream, Warren Whittaker, his long-time collaborator, finally abandoned map dowsing and advanced a new argument for another target near the Abrolhos Islands. The account was based on the diary maintained by von Malapert. According to Whittaker (2000),

“These “logs” (i.e., written records from the German survivors) contain clear evidence that the battle actually took place west of the Abrolhos Islands and not in the northern or Detmers area. The Abrolhos Islands site is consistent with KDLS Target No. 3 (suspected site of the wreck of HSK Kormoran) at 28°39′S 113°22′E; Error = 196 NM) (Whittaker, 2000).

The claim advanced by Whittaker in 2000 was contradicted by the fact that both Meyer, the Kormoran navigator, and von Malapert, specified the approximate distance covered by their lifeboat over the *entire* voyage, and they put the distance at 150 miles and 153 miles respectively, about half of the distance from the Houtmann Abrolhos Islands to Cape Cuvier by sea.

Whittaker was not the last person to focus on interpretation of the diary. The first endorsement came from LCDR David McDonald RAN. McDonald reviewed and distributed an analysis of the lifeboat voyage that placed the point of origin in a large ellipse off the coast in the latitude of Port Gregory, the latitude long-advocated by his mother on the basis of the oral history accounts described below (McDonald, 2003).

The second endorsement came from the RAN Seapower Centre (Johnstone et al., 2003). The RAN conducted a Lifeboat workshop in order to facilitate recognition of the site of the wrecks. An expert panel was formed to resolve the issue. The panel's conclusions were as follows:

“Analysis of the lifeboat voyage by the workshop panel suggests that the correct site of the battle between SYDNEY and KORMORAN lies between the Whittaker and Detmers positions. However, given the paucity of information from the lifeboat log, limited meteorological data from November 1941, and unclear data on the handling characteristics of the lifeboat, the actual position of the battle cannot be narrowed sufficiently to confidently suggest the resting place of the KORMORAN wreck. For these reasons also the Detmers and the Whittaker positions cannot be definitively ruled out at this time.”

Consideration of the oceanographic evidence removed the Abrolhos arguments from the table, rendered in-shore locations improbable, and indicated that the discipline could not be used to provide an *accurate* or *efficient* solution.

## Historical analyses

In the first professional historical analysis of the engagement, Herman Gill located the contact and battle positions for *Kormoran* and *Sydney* near 26°34′S 111°E and 26°40′S 110°33′E, respectively, the second of these positions being 42 NM from the wreck. Gill was not of course touched by any interest in a search for the wrecks. As discussed above, Barbara Winter interpreted Detmers' Diary correctly, and located the battle and therefore the wrecks in the vicinity of 26°S 111°E (Winter, 1984, 1991).

The next historical analysis was published by Wes Olson in 2000. Based on the map on page 192 (Olson, 2000), Olson located *Kormoran* near 26°34′S 111°E when she first encountered *Sydney* on November 19th, and the extrapolation developed by Olson placed the battle and therefore the wrecks near ~ 26°42′S 110°35′E, 42 NM from the wreck of *Kormoran*.

The final historical analysis, by Olson et al. (2001), reverted to the argument advocated by Winter (1984); Winter (1991), however their paper included a new and independent detail. They assumed that 26°34′S 111°E specified *Kormoran's* noon position, and used dead reckoning based on Detmers' account of *Kormoran's* subsequent movements to locate the wreck near 25°58′S 110°56′E. This position is 11 NM from the wreck of Kormoran, however, as they advocated a search circle with a radius of 10 NM only, their analysis did not quite include the wreck of *Kormoran*.

The historical analyses focused almost exclusively on the content and interpretation of the report included in Detmers Battle Summary. Before turning to the cognitive analyses, brief consideration will be given to the technologies that so captured the attention of the WAMM, the RAN, and the public agencies engaged in the search between 1981 and 2005.

The original historical argument provided a target accurate to only 30′ or 27–30 NM, thereby defining an area of ~ 3400 SNM. When combined with dead reckoning a more specific target was provided but in each case the solution relied on only one report and one source, a source that had provided inaccurate and inconsistent information at the time of the in-water search after the engagement.

## Magnetic anomalies, map dowsing, and oral history

The amount of credence placed on Montgomery's claim by the WAMM is evident in the fact that the relevant team used it to justify an in-water search involving collaboration with the RAN while searching to the south of 27° South, more than 130 nm to the south of the position actually recommended by Montgomery, and the position recommended by Winter, and the wreck of Kormoran (See Green et al., 1984).

The Map Dowsing argument passed through two incarnations. The first of these involved traditional hand-based map dowsing or “divining” while the second was based on the principle of Electron Spin Resonance. Each of these procedures pointed to positions off the Abrolhos Islands, nearly 200 NM from the now known positions of the wrecks. One of the positions allegedly attracted a search from an RAN submarine in 2000. In 2003, based on Whittaker's interpretation of von Malapert's diary of the voyage of a lifeboat, the RAN established a workshop involving four senior navigators, and provided qualified endorsement for the navigation argument for the Abrolhos Islands.

The Oral History argument reflected an interesting and recently established branch of history, however it is usually used to capture *subjective* experience as distinct from fine details about the timing or the location of specific events. For example, Studs Terkel, a key player in the tortuous history of the discipline, noted that,

“They would sit around and tell us their hard luck story. Whether it was true or not, we never questioned it. It's very important you learn people as they are. At that particular moment when you are talkin' to that person, maybe that's how that person were. Tomorrow they can be different people.” (Emma Tiller, *a cook in Western Texas, as reported by Studs Terkel, 1970)*.

In fact, the majority of the oral history reports submitted as evidence of a battle near the Abrolhos Islands involved eye-witness accounts by individuals, and the claim that they involved Oral History was therefore problematic. As accounts based on Remote Memory, they involved critical flaws. For example, Bryan Clark, the journalist who first recorded many of the stories in the late 1980's, opined that some of them at least reflected experiences from later years, when Catalina maritime patrol aircraft flew practice missions off Port Gregory. In our view (Kirsner and Dunn, 1998c), the accuracy levels for recall of remote events are so low after an interval of nearly 50 years that little confidence can be placed in them (e.g., Wagenaar, 1986). Furthermore, the historian providing advice to the *Joint Standing Committee on Foreign Affairs, Defence, and Trade* (JSCFADAT) advised that few of the accounts included any form of link to the engagement between *Kormoran* and *Sydney*. Statistical analysis supported this argument and revealed that fewer than 10% of the accounts actually included any link with *Kormoran* and *Sydney* at all. Another line of argument indicated that the reports emerged from positions covering more than 20,000 SNM, hardly a pointer to a specific battle on a given day. None of these arguments prevailed. The JSCFADAT gave the oral history argument pride of place for the 2001 Shipwreck Seminar; and the RAN and the RAAF implemented expensive and risky aerial and surface searches of the target positions identified by the oral historian.

Analysis implemented by the author and his colleagues rejected the remote memory or “oral history” argument, despite strong support from WAMM and the JSCFADAT (Kirsner and Dunn, 1998c), and the map dowsing case was intrinsically weak (Kirsner and Dunn, 1998b).

## The cognitive sciences

### Review of wreck-hunting

Following the Oceanography Workshop, and aware that oceanography would not be able to provide a precise target, the author sought to achieve a better understanding of the challenges and solutions associated with deep-sea wreck-hunting. The first section involved a review of the available evidence on deep-sea/off-shore shipwreck hunting, with the focus on the identification of search targets and the definition of search areas. The critical history of the engagement between *Kormoran* and *Sydney* was published by Hermon Gill in the first book of his two volume history of the RAN in World War II (Gill, 1957, 1985). Gill wrote more than 12 pages about the engagement between *Sydney* and *Kormoran*, however, unavoidably, his analysis was based exclusively on reports provided by the *Kormoran* crew, and that was perhaps the first trigger for doubt among the old salts in the local community. Further doubt about the reliability of the reports provided by the *Kormoran* survivors was facilitated by the fact that the Captain and the Navigator provided inconsistent reports to the RAN officers during late November and early December 1941. A second cause for doubt arguably involved the gradual release of information about the role of Signal Intelligence following World War II, a process that was still yielding new information and an occasional surprise up to the very end of the twentieth century (e.g., Fry, 2012). A third issue that compromised the search debate between 1991 and 2013 involved the widespread assumption by the official bodies associated with the search that a sou'wester was the key to expertise, and that scientists without sou'westers had no business entering the arena.

The first section of the review involved consideration of five examples of deep-sea wreck-hunting. The first example involved the search for the wreck of the *Titanic*, sunk on April 14th–15th 1912. The search area adopted for the first three searches for *Titanic* appear to have been constrained exclusively by navigational reports about the position of the sinking, and the resulting search involved only 100 SNM. In 1985 the area was expanded by Ballard to 150 SNM to incorporate the southerly movement of the lifeboats between the sinking and the rescue but even here the critical factor involved navigational reports about the final position of the lifeboats (i.e., without reference to oceanographic assumptions), coupled with a decision to commence the search beyond even that position, and shape the in-water search from that position toward the estimated position of the wreck of *Titanic*. In summary, the critical points were determined solely by navigational reports although the reports were selected to define a search area that reflected the movement of lifeboats in the water. The current in-water technology enables more efficient in-water search, but that should not be critical if the actual search box has been chosen with due consideration for uncertainty.

The second example involved the German battleship *Bismarck*, sunk on May 26th 1941. The search area for *Bismarck* was shaped around reports about the sinking position provided by British battleships HMS *King George V* and HMS *Rodney*, and British cruiser HMS *Dorsetshire*, although only the third of these was present when *Bismarck* actually sank. As the search unfolded however the focus shifted to a search for debris, and then a landslide on an underwater mountain, the end of which finally revealed the location of the wreck. The assumption adopted by Ballard was that the landslide was actually triggered by *Bismarck*, when it hit the ocean floor. Ballard indicated that the search area involved was = 200 SNM. Descriptions of the search operations for these wrecks are detailed in Ballard (2008), and available from earlier reports by Ballard (1988, 1990); Ballard and Archbold (1999).

The third example involved the US Aircraft Carrier *Yorktown*, lost during the Battle of Midway. A review of the search indicated that a search area of ≤ 500 SNM was used by Ballard, and that the area was specifically extended to the south in order to cater for uncertainty about the distance covered by Yorktown between the final aerial attack on the afternoon of June 5th and her sinking on the morning of June 7th following a submarine attack on June 6th.

The fourth example involved the search by David Mearns for the bulk carrier *Derbyshire*. Initial analysis revealed three reliable reports of oil slicks. Further, analysis suggested that the wreck might be up to five nm to the north of the position where the oil actually breached the surface. Mearns (1995) defined two search areas, of ~ 90 and ~ 170 SNM as “high” and “low” probability areas respectively, and the wreck was duly found in the predicted area. It is a matter of interest that Mearns used “the principles of modern probability analysis” as described by Discenza and Greer (1994) to shape the search plan.

The fifth example involved the search by Mearns for the wreck of HMS *Hood*, sunk on May 24th, 1941. Information about this search was not available in the public domain until 2001, and the work did not therefore inform the author's review. As described by Mearns and White (2001) however, the record included no fewer than 10 reports about the location of *Hood*. Mearns rejected three of these because they depended on aerial calculation. Of the remaining seven no fewer than five were from battleships or cruisers and occupied a very tight box of approximately 40 SNM. The remaining two involved positions determined by destroyers and either dead reckoning or movement after a substantial time lag (and therefore uncertainty over wind and current). Mearns tabled two search boxes for operational purposes, of ~ 600 and 200 SNM respectively, however the quantitative bases for these areas remain unspecified. Mearns and White (2001, p. 107) noted however that,

“The first two decisions were dictated by the simple application of the navigational errors we had found to exist in the reported sinking positions of the reported sinking positions during the First and Second World Wars. The errors that I chose to apply in this case were divided into two different categories: the worst error reported by a surface ship and the average error reported by a number of surface ships. These circles of error were drawn around each of the five most likely sites for Hood to have sunk.”

The details of the in-water searches conducted by Mearns have not been published, as they formed “part of a commercial operation.”

The history-based procedures implemented by Ballard and Mearns realized substantially smaller search areas than those generated by the 1991 Oceanography Workshop. The three searches by Ballard involved areas that ranged 150–500 SNM, values dramatically smaller than those generated by the 1991 Oceanography Workshop, and areas that enabled discovery of each of the wrecks concerned. The areas used by Mearns in searches for the SS *Derbyshire* and HMS *Hood* are less clear but they were probably less than 600 SNM, and they too relied primarily on contemporary reports from observers.

The review removed any doubt about the relative merits of the oceanography-based and history-based procedures in research to define accurate and efficient areas for in-water search. The oceanography-based procedures yielded an overall area of ~8400 SNM for *Kormoran* [sum of areas provided by Hughes (1991) and Steedman and McCormack (1991)], although even that solution came with a significant caveat from CSIRO based expert Alan Pearce. The central issue was therefore clear. As the oceanography-based analyses for *Kormoran* had produced search areas between 10 and 100 times larger than the areas used for the *Titanic, Bismarck*, and *Yorktown* searches, an historically-based analysis was essential, and the author embarked on the collation and analysis of the survivors' reports.

The review indicated that search definition was dominated by reports from captains, navigating officers and professional observers, and that it generally resulted in areas of 500 SNM or less.

### The Kormoran database

The critical question concerned the scope, extent, and reliability of the reports provided by the German survivors. Given inconsistent reports from the Captain and the Navigator, was it possible to accept as valid reports from other crew members, particularly if they too varied from report to report? The records at WAMM provided an initial set to work on, and the books published by Montgomery (1981) and Winter (1984) provided pointers to additional material, however it was by no means obvious that these sources covered the full extent of the reports provided by the Kormoran survivors and the RN/RAN interviews and interrogations.

The second step involved archival research in London, Washington, and Norfolk, Virginia as well as Sydney, Perth, Canberra, and Melbourne, in Australia. When combined with the material available from Fremantle, the archival research yielded a total of 73 reports that involved reference to the absolute or relative location of *Kormoran*, a further nine about the bearing and distance of *Sydney* relative to *Kormoran* for the period between the battle and the last sighting of *Sydney*, and a further 44 that involved official or unofficial reports from RN or RAN officers. Collation of the reports and the creation and analysis of a substantial database located the project firmly within the tradition of error analysis in Cognitive Psychology and Human Factors. The project therefore required consideration of two data types, involving the positions of objects in the ocean and the reports of the survivors, and three methodological approaches, involving oceanographic hindcasting, historical review, and cognitive analysis and modeling.

The products of the archival research were summarized in the following extract (Kirsner, 1997b). The paper was entitled *The War of the Ghosts: Using dusty records to hind-cast the locations of HMAS Sydney and HSK Kormoran* and it was presented to a Humanities Conference at the University of Western Australia.

The traditional problem with archives is that they contain too little information, and that too many inferences must therefore be left to logical analysis or intuition. The archives concerning the loss of *Sydney* and *Kormoran* arguably involved the opposite problem where location is concerned. Analysis of the archives and other historical sources revealed at least 60 separate sources of information about the location or locations of the wrecks, and these sources identified no fewer than 25 different sites, only a few of which could be discounted absolutely. The sources are, furthermore, distributed among five or six layers involving SAR operations, the interrogation of survivors both during and after the SAR operation, operational reports prepared by RN and RAN officers, administrative reports, political reports and, finally, historical argument. Worse still, the deeper layers even include reports suggesting new sites, not recorded in the earlier reports.

The data depicted in Table 1 is a summary of the reports from the Kormoran Database. The reports were obtained from numerous sources. Some of these were available from Montgomery (1981) and Winter (1984); some were from the library of the West Australian Maritime Museum; some were obtained from the state archives in Perth, Melbourne, Sydney, and Canberra; and a handful were discovered in the national archives of the UK and the USA, and two were discovered by Hore and Mearns in the Old Admiralty Library in London. Most of the reports were collated between 1993 and 1997, however additional items were added later as they became available. A summary file was provided to the Cole Commission at its request in 2008, and re-distributed by it on request.

**Table 1:** **Stem and leaf plot of Reports from Kormoran survivors**.

**Type of Report**	**AR**	**E**	**Comment**
1.26°S 111°E (to be read as ±30′)	17		Mode (including one report from Bunjes, two from Detmers)
• 26°S 110°E		2	EC including one report from Detmers
• 26°S		1	EO
• 26°S 11°E		1	EO
• 26°S 108°E		1	EC
• 26°S 111°40′E		1	EC
• 26°S 111°21′E		1	EC
• 24°S 111°E		1	EC
• 25°S 111°E		2	EC including one report from Detmers
1a. 26°S 111°E (to be read as ±30′)	1		Meyer as revealed by diary in 2000
• 27°S 111°E		5	Initial reports from Meyer
• 26°30′S 111°40′E		1	Later report from Meyer
2.120 nm from Coast	4		Bunjes; 120 nm selected on basis of MDP
• 150 nm from coast		2	EC
• 60 nm off land		1	EC
3.160 nm SW of Cape Cuvier	0		Bunjes; Cape Cuvier selected on basis of MDP
° 160 nm SW of *NW Cape*		1	
4. Geraldton signal 2 (gap) 7S 11115E	1		26°07′S 111°15′E selected on basis of MDP
° 7C 115E 1000 GMT		1	
5. Sailed 150 nm NE to land	1		Meyer—lifeboat diary
• Sailed 153 nm NE to land		1	V Malapert—lifeboat diary
6.26°34′S 111°E 3	6		Detmers: Winter (1991) classified as *noon* report
• 26°32′S 111°E		6	Detmers
• 25°34′S 111°E		2	Detmers
• 26°31′S 111°E		1	Detmers
7.130 nm SW of Shark Bay	4		Habben
8. Due West of Shark Bay at 2000 h	1		Detmers to be “due west of Shark Bay at 2000”
° 120 nm SW of Fremantle		4	EC
° 100 nm off Fremantle		1	EC
° 130 nm due West of Perth		1	EC
° 125 degrees SW of Frem.		1	EC
° 20 nm SW of Fremantle		1	EC
Total	35	38	

*The numbered and colored items define the stems. The bulleted items comprise the leaves associated with each stem. The gray items are “pure” errors*.

Table 1 can be read as a form of “stem and leaf” diagram. The numbered reports in the fawn rows were included in the final analyses; the reports with black bullets were treated as derivatives, and discarded; and the bullets with open circles were treated as outright errors.

The Kormoran Database comprised more than 70 reports by survivors about the location of the wreck of Kormoran, a source of evidence that would be invaluable for an accurate and efficient solution provided that the major part of the database was reliable. A substantial database was essential if the solution was to be efficient as well as accurate.

### Reliability of the Kormoran database

**Figure 4:**
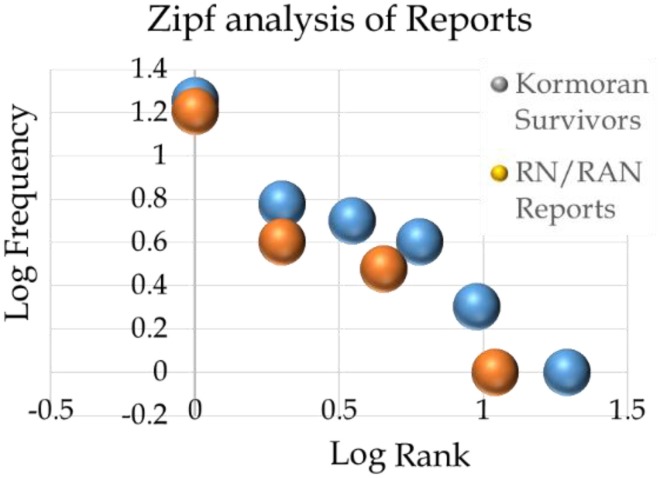
**Zipf functions for the Kormoran survivors (blue circles) and RN/RAN officers (red circles) associated with the 1941 interrogations and interviews**. The figure depicts Log Frequency plotted against Log Rank for each set of data.

Figure 4 is a plot of the data from Table 1. The axes depict the data in Log-Log coordinates. The y-axis reflects a log transformation of the number of reports associated with each Type of Report. The x-axis reflects a log transformation of the rank value of each Type of Report. Following Zipf (1949), the resulting function is linear and the observed pattern is consistent with the proposition that resource limitations played a role in report selection and recall. Zipf demonstrated that a linear relationship is observed for many relationships provided that Log-Log axes are used. The *r*^2^-value for the survivors' reports was 0.89, a value that accounts for more than 80% of the observed variation in the data. A small sample of the many variables that honor Zipf's Law includes word frequency distributions for English (Zipf, 1949; Miller and Newman, 1958), recall (Kaplan and Carvellas, 1969), and character frequency distributions for Japanese Kanji (Speelman and Kirsner, 2005). The figure also depicts the equivalent set of results for the 44 reports tabled by RN and RAN officers, and it shows essentially the same pattern.

Zipf's Law does not constitute a “proof” in the definitive sense of that term. Rather, it is a pattern we would expect to observe in a memory study involving recall of a number of words. The distributions of the reports are consistent with the proposition that the observed patterns reflected randomly distributed memory or transcription errors. The most obvious alternative hypothesis involved the argument that the Kormoran survivors rehearsed their answers. In the extreme case, this approach would have produced just one Type of Report, or something close to that. The fact that the reports were distributed across eight or more than eight referents contributed to the assumption of reliability.

Two issues were critical to the final assessment; first, the fact that no fewer than eight independent groups of reports or *constraints* pointed toward the same general position, and, second, the fact that all five lifeboats either arrived at or were approaching one point on the coast when they were discovered by rescue craft, a degree of convergence that would have been improbable had the survivors had no idea where they were, or where they were going.

Mathematical analyses determined that the Database was reliable, overall, although it was clear that a number of individual reports were not.

### The 2004 solution

The majority of the reports used for the wreck-hunts described in the review relied on reports from navigators about the coordinates of the vessel prior to or at the moment of loss. Each set of reports involved some variability among the navigators who reported the loss of a vessel, involving the own ship navigation, time of the observation, or the position of the survivors, however they all relied on professional reports, where precision was reduced because each navigator produced a unique solution. The *Kormoran Database* reflected a very different form of evidence and error. Many of the reports provided only a *general* guide to the location of *Kormoran* at the time of her loss, for sailors at sea, and at risk, and an alternative approach therefore involved a weaker assumption that all or at least many of the reports were valid, and could therefore be considered as a set to point to the position of the wreck. Working alone in the first instance, and then in collaboration with John Dunn, this approach was refined in four stages. The stages were as follows:

1. Discount and remove obvious errors from consideration.
2. Group reports that involved a single concept, or “root,” in evolutionary terms.
3. Develop principles to resolve competition when reports in a single group involved inconsistent evidence.
4. Design and implement a mathematical decision model to integrate the surviving statements or constraints, the task completed by John Dunn.

#### The constraints

The overall procedure was designed to produce a single and accurate estimate of the location of the wreck of *Kormoran*. The analysis evolved over the period 1991–2004. The concept of *converging operations* shaped the research.

##### Constraint 1: 26°S 111°E

The majority of the 18 reports that involved 26°S 111°E were provided by Wireless Telegraphy Officers (WTOs), adding further weight to the validity of the report. The critical weakness with the mode is that the position as reported, 26°S 111°E, is accurate to only the nearest degree, and for wreck-hunting purposes it should therefore have read 26° ± 30′S 111° ± 30′E, where provision for error identified a search area of 3400 SNM.

##### Constraint 2: report by bunjes that the battle occurred “120 nm from the coast”

The second constraint involved the distance from the coast. Three estimates were available from the reports from the Kormoran survivors, at 60, 120, and 150 nm. One hundred twenty nanometer was adopted for two reasons: First, Bunjes provided 120 nm value on three occasions during the fortnight after the battle whereas he provided the value of 150 nm in one report only, and years after the event; and, second, 120 nm provided a better fit with the first and third constraints under the Minimum Distance Principle described below.

##### Constraint 3: 160 nm SW of NW Cape (interpreted as Cape Cuvier)

The third constraint, also attributed to Bunjes, involved the report that the battle occurred 160 nm “South-West of North-West Cape.” North-West Cape is more than 300 nm from the area of the battle, and out of the game. An error is the obvious explanation but what sort of error. While the author was working out the tracks of the lifeboats in 1991 (Kirsner, 1991), detailed analysis indicated that all five of the lifeboats could have been heading for the same position on the coast. Two of the lifeboats—those captained by Meyer and Kohn–beached 5 and 12 nm north of Cape Cuvier respectively, and the other three were sailing east and more and less directly for Cape Cuvier after some 4 or 5 days drifting to the north with the current and wind.

### Triangulation

**Figure 5:**
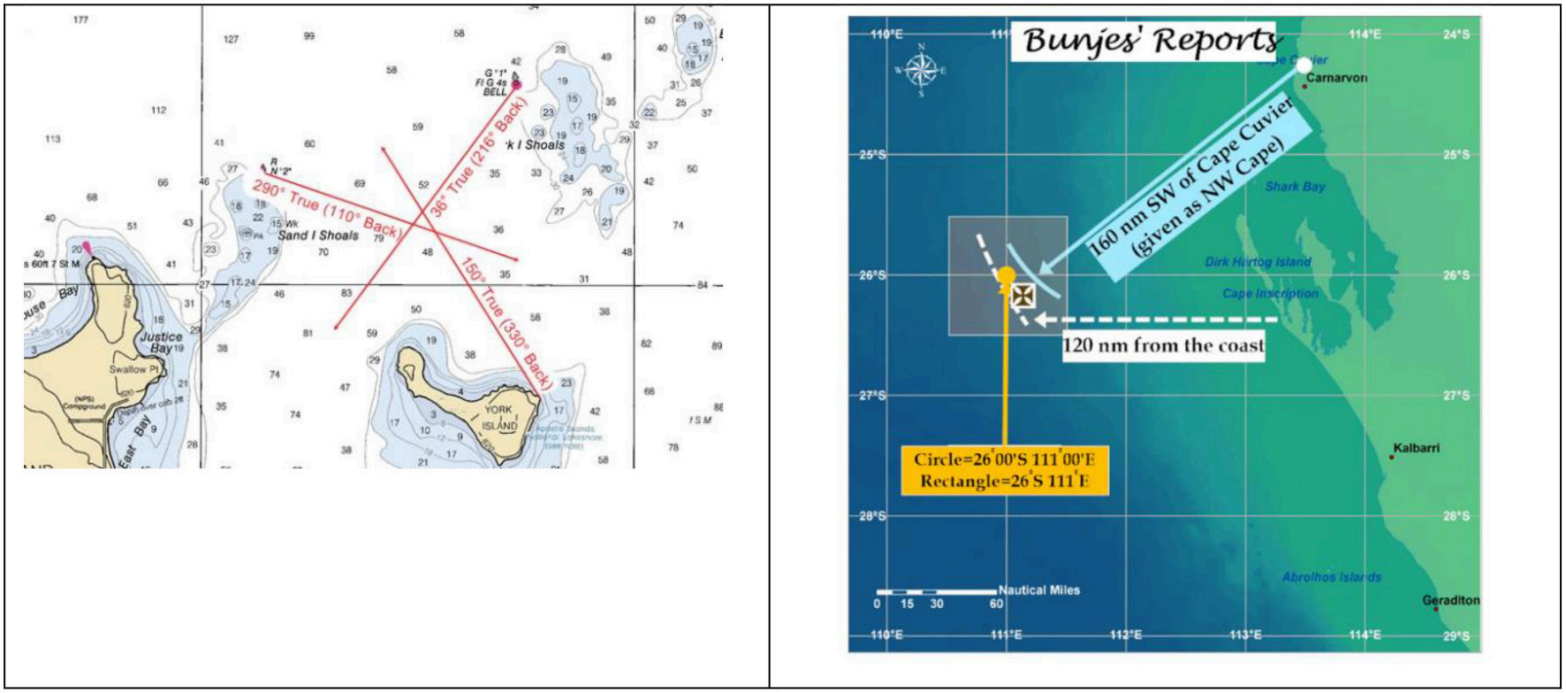
**(A)** Illustration of triangulation as the term is generally understood by mariners (Hansel, 2011). **(B)** Convergence among reports from Bunjes. The circle and the rectangle represent 26°00′S 111°00′E, and 26°S 111°E, respectively.

Given the availability of multiple and converging constraints, triangulation provided an appropriate model for our approach to the problem. Maritime triangulation is illustrated in Figure 5A. In that example the approximate location of a ship is assumed to be inside the triangle defined by convergence among the three observations or “Lines of Position” specified by the navigator. Figure 5B is a summary of three reports provided by Wilhelm Bunjes, a sometime officer in the pre-war German merchant marine. Argument for the reliability of Bunjes' intentions came from the fact that one of his reports about the Kormoran officer's was “masked” in the archives for nearly 30 years, allegedly to protect him from repercussions associated with his anti-Nazi sentiments.

Although the Kormoran Database included reports that relied on a variety of referents, including cartographic coordinates, distance from the coast, and distance and bearing from coastal features, it is evident that Bunjes' reports converged on a single “position,” a position that could be used to define the point of disembarkation from Kormoran prior to her sinking. Convergence was not straightforward. As indicated above in regard to the first three constraints, convergence was achieved only when Cape Cuvier was substituted for North-West Cape as the coastal feature, and 120 NM was adopted in preference to 150 and 60 NM as the distance from the coast, and even then the “error” associated with the first constraint a significant handicap.

The authors' use of triangulation is closer to its nautical roots than it is to the role of triangulation in the social sciences (e.g., Yeasmin, 2012). In the latter case it constitutes a form of validation although it may also be implemented in order to increase “understanding” of a specific problem. In the present case however, triangulation is being used to refine the location of a wreck by using “Lines of Position” when most if not all of the lines involve an element of potential but unknown error and uncertainty.

The approach outlined above involves very different principles and assumptions from the oceanographic models, however it is the contrast with the historical analyses that is particularly interesting. Six historians or teams of historians tabled solutions to the Search Definition problem; Gill (1957, 1985); (Error = 42 NM), Winter (1984); Winter (Error = 7 NM), Winter (1991); Winter (Error = 7 NM), Olson (2000); Olson (Error = 42 nm), Olson et al. (2001, Error = 11 nm), and Hore and Mearns (2003); Hore and Mearns (Error = 7 NM), and in each case attention was restricted to a single option or interpretation of one or possibly two reports. It is evident that the attention of the historians was focused either substantially or exclusively on the reports provided by Detmers, the Captain of *Kormoran*, and that the only issue that vexed them concerned the relative merits of the *noon* and *battle* interpretations of 26°34′S 111°E. Indeed the only individuals or teams to opt for that remote position as the position of the *battle* and therefore the wrecks were Gill (1957, 1985), Olson (2000), and Mearns (e.g., Finding Sydney Foundation, 2007), and Mearns included the so-called *noon* position, 26°34′S 111°E, in the in-water search area in 2008, a decision that depended on his recommendation alone.

####  

##### Constraint 4: 2#°#7′S 111°15′E; geraldton signal received at 1800G (interpreted as 26°07′S 111°15′E)

The Geraldton signal has come down to us in two forms. The first form was included in a report prepared by SWACH and dated November 27th. The wording of the report is as follows:

“Geraldton radio reports that at 1005Z/19/11 they received a weak message. The beginning was unintelligible. Then followed “7C 115E 1000 GMT.” The radio operator could not estimate the distance. No Qs were distinguished. They waited 2 min but there was no repetition”

The second version of the report is included in the Fremantle Report of Operations for the period November 24–29th (see Olson et al., 2001, p. 38). The wording of this report is as follows:

“At about the same time Geraldton radio picked up a weak signal unintelligible excerpt for '2 (gap) 7 111 15 East 1000 GMT (These two reports were not received until 1345H/27)”

The number of operational and cognitive steps between the Kormoran transmission and the SWACH report of operations is difficult to estimate. Radio signals occurred in noisy environments, and it is no accident that signal detection theory (Tanner and Swets, 1954) evolved as a response to the classification problems experienced by radio operators during and after World War II (e.g., Shannon, 1949). We can safely assume that the radio operator in Geraldton was dealing with a noisy signal. She or he may have misheard parts of the signal. They may have heard it correctly but made a transcription error. They may have transcribed the signal correctly, only to have a supervisor introduce an error, in reading or during preparation of a signal for transmission to SWACH. We do not know for example why the second report comprised “2 (gap) 7” whereas the first comprised “7C,” and we probably never will.

### The minimum distance principle

**Figure 6:**
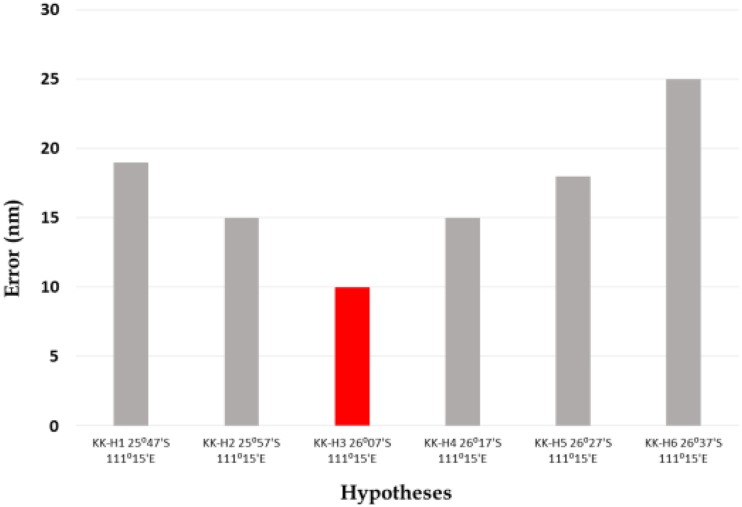
**The Minimum Distance Principle**. Distance between six candidate interpretations of the signal and the position defined by the first three constraints.

The solution adopted to solve the uncertainty associated with this potential constraint involved the Minimum Distance Principle. In brief, six alternative interpretations of the signal were benchmarked against the established candidates; that is, constraints 1, 2, and 3, and the alternative that involved the smallest movement was adopted. The positions in the mix were; 25°37′S 111°15′E, 25°47′S 111°15′E, 25°57′S 111°15′E, 26°07′S 111°15′E, 26°17′S 111°15′E, and 26°27′S 111°15′E. As illustrated in Figure 6, the fourth of these positions provided the best fit, and 26°07′S 111°15′E was therefore adopted as the fourth constraint.

####  

##### Constraint 5: meyer's “lifeboat originated 150–153 nm SW of landing point”

The critical information is summarized in Section Oceanographic and Navigation Analyses, The Navigation Argument. The uncertainty associated with the relevant estimate was acknowledged by Meyer.

##### Constraint 6: report from detmers' battle summary: 26°34′S 111°east

Barbara Winter provided the critical interpretation of Detmers' Battle Summary (Winter, 1991). Her analysis left no doubt that 26°34′S 111°E and 26°S111°E were the *noon* and *battle* positions of *Kormoran*, respectively.

We nevertheless used the distance between the solution offered by this potential constraint and the position provided by other constraints to test the *noon* and *battle* interpretations of Detmers' report. The *noon* interpretation won that competition too, and we therefore adopted the *noon* interpretation for integration purposes. Our dead reckoning analysis confirmed the argument advanced by Olson et al., 2001, and yielded a solution one NM to the North of theirs, 12 NM from the wreck of Kormoran.

##### Constraint 7: report by Habben: “130 nautical miles South-West of Shark Bay”

Siebelt Habben, a medical doctor, was repatriated to Germany in 1943 as part of a Prisoner-of-War exchange. Habben provided descriptions of the action between Kormoran and Sydney to the German naval authorities, and the Kreigsmarine subsequently included them in Operationen and Taktiks, Volume 10.

##### Constraint 8: detmers' statement that Kormoran should be due west of Shark Bay at 2000G

According to Detmers (1959),

“The KORMORAN was proceeding at medium speed on her usual sweep and gradually approaching Shark's Bay from the south west. At 1500 h I checked the ship's course and decided to carry on without change until 2000 h, and then turn eastward toward Shark's Bay.”

This solution to this constraint also involved dead reckoning, from the assumed track of Kormoran from noon to 1700 h.

##### Constraint 9: mathematical analysis identified a “circle of equal speed” for the life-rafts discovered by aquitania and trocas

This analysis was submitted to and published by the 2001 Shipwreck Workshop (Dunn and Kirsner, 2001). The mathematical model designed by Dunn was based on three assumptions about the life-rafts;

- They were under the influence of the same currents and winds, and they would therefore display essentially the same “sailing” characteristics.
- They had similar buoyancy and “sailing” characteristics, and they would therefore move downwind in a similar direction and at a similar velocity.
- They would conform to wind direction ±35°, an assumption accepted by the Search and Rescue profession.

**Figure 7:**
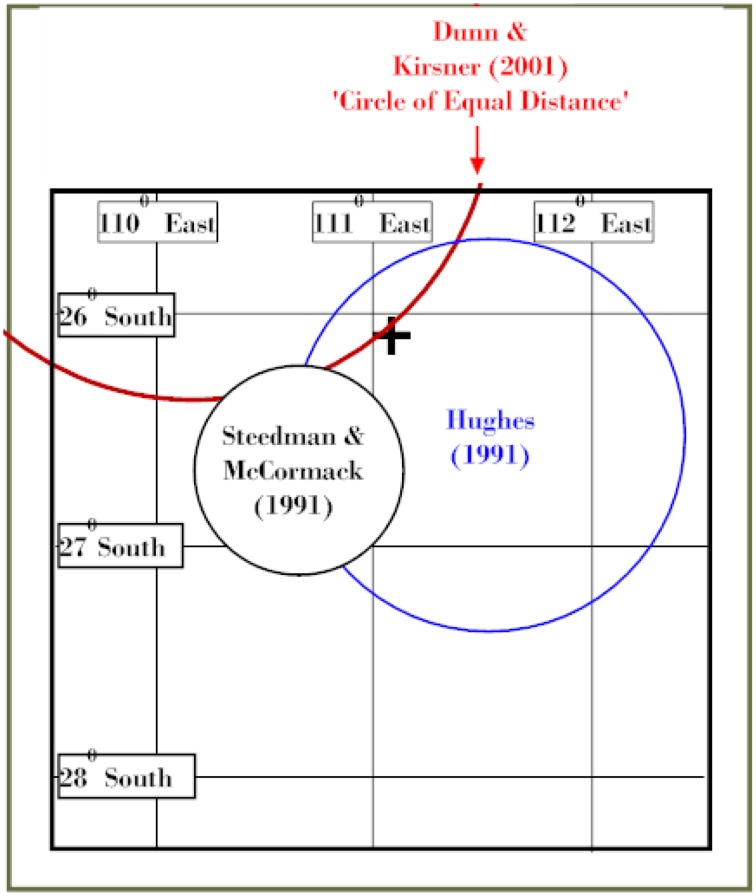
**Circle of Equal Distance (Figure from Dunn and Kirsner, 2001)**.

The cross in Figure 7 denotes the now known position of *Kormoran*. The distance between Kormoran and the nearest point on the circle is ~2 nm. The blue circles denote the areas advanced at the 1991 Oceanography Workshop by Hughes (1991) and Steedman and McCormack (1991). The Circle of Equal Distance reflected a purely mathematical solution based only on the assumption that the life-rafts were influenced by the same forces, and drifted at the same velocity. The area of the red circle is irrelevant; prediction involved the circumference.

#### Integration

In 2004 John Dunn designed a mathematical generalization of the Minimal Distance Principle. The aim of the generalization was to identify the most likely position of the wreck, and the procedure involved selection of the position that involved the smallest “movement” for the set of nine constraints outlined above. We therefore integrated all of the available information under the assumption that each piece of information would be broadly consistent with the remainder, and that integration would converge on the most likely point.

For each candidate location, corresponding to a point in the ocean, and each constraint, we calculated the minimum distance that the candidate location would have to be moved in order to satisfy the constraint exactly. We referred to this measure as the *error distance* for each location-constraint pair. We then calculated the *average error* distance across the set of constraints for each location which then provided a single *goodness of fit measure* for that location. Clearly, a candidate location with a relatively small average *error distance* satisfies the constraints to a greater extent than a point with a relatively large average *error distance*. No single candidate location satisfied all of the constraints exactly.

Averaging the error distances treats each constraint as having the same weight or importance. We considered and rejected a range of weighting schemes, however we were not persuaded that there was any basis for treating one constraint as more critical than another. We were also guided by studies of expert decision making in which equally weighted linear models (so-called improper linear models) are nearly as efficient as optimally weighted models (Dawes, 1979).

Integration yielded 26°04′S 111°02′E as the position of the wreck. This position is 2.7 nm from the true position of the wreck as established by the *FSF* in 2008 (Finding Sydney Foundation, 2008). The approach was described by Kirsner and Dunn (2004) and Dunn and Kirsner (2011). The recommendation was also used and published by the FSF in 2005 and 2007.

#### Performance

##### Accuracy

**Figure 8:**
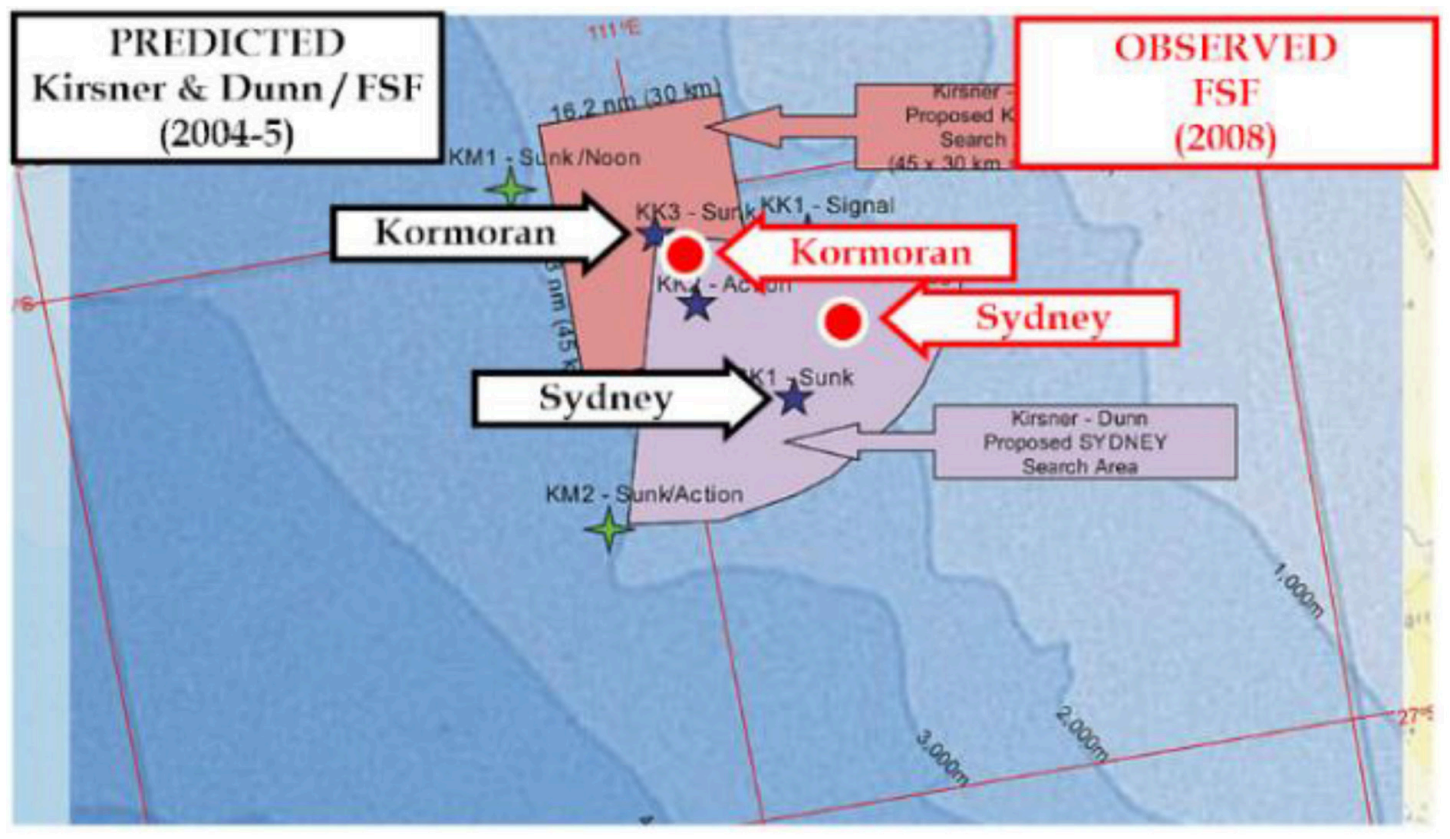
**Map including search areas prepared by Bob King for use by FSF in 2005**. The figure compares the predicted and observed positions of the wrecks Finding Sydney Foundation (2005).

FSF Director Bob King chaired the Technical Search Committee of the FSF from 2005 to 2007 inclusive. In 2005 King designed a Powerpoint presentation for use by the FSF. The critical figure is reproduced as Figure 8 below. The figure includes the positions recommended for *Kormoran* and *Sydney* by the FSF in 2005 on the basis of the arguments and recommendations advanced by Kirsner and Dunn (2004). They are depicted as black stars (from the original) identified by the black labels (added) indicating the names of the two ships. The now known positions of the two wrecks are depicted by solid red circles identified by red labels, each of which has been added to the map. The errors for the two recommendations were 3 and 9 NM for *Kormoran* and *Sydney* respectively. Research and argument advanced subsequent to that date was superfluous, and served only to transfer responsibility for the success of the search.

##### Efficiency

The pink rectangle and the purple quadrant indicate the search areas recommended by the FSF for *Kormoran* and *Sydney* respectively, in 2005. The area of the pink rectangle, the recommended search box for *Kormoran*, reflected conventional statistical analysis based on the latitude and longitude values associated with each of the nine constraints. The area was therefore defined by the 95% confidence intervals for the x (longitude) and y (latitude) values based on positions attributed to each of the nine constraints. The area of the rectangle, 400 SNM, and the location of the wreck of *Kormoran*, can be compared with the area of 2200 SNM adopted by the FSF on the advice of Mearns a few weeks before the in-water search in 2008.

##### Explanatory power

The final cognitive analysis reflected the majority of the data summarized in Table 1. The cognitive solution was, furthermore, consistent with the known tracks of *Sydney* through the area, and the oceanographic solutions described above although these considerations did not contribute directly to the quantitative solution. The historical analyses exploited only the report originally extracted by Winter from Detmers' Diary (Winter, 1991), based on the noon position plus dead reckoning.

The research provided an accurate estimate of the position of the wreck of Kormoran, an efficient solution given a search box of < 400 SNM, and it exploited more than 50% of the items in the Kormoran Database. The research also provided an accurate estimate of the location of *Sydney*, based on a time series analysis of her reported bearing and distance from Kormoran over a 5 h period after the battle.

#### Opportunity cost

The author did not review information about the 1968 search for the USS *Scorpion* prior to creation of the Kormoran Database. However, in 2006, when the FSF invited John Dunn and the author to table a new search proposal, we revisited the Search Definition problem, gave consideration to the Baysian description of the search for Scorpion (See Sontag and Drew, 1999), and tabled a new proposal that included provision for expert-based weighting for the individual constraints.

## Skill acquisition

Table 2 is a summary of the recommendations advanced by the author and his colleagues between 1991 and 2005. The Search Definition problem was solved by Australian science for both Kormoran and Sydney by 2005.

**Table 2:** **Summary of positions advanced by the author and his colleagues for Kormoran**.

**Sources**	**Coordinates**	**Error (NM)**
**STAGE 1: OCEANOGRAPHY/SAR**		
Kirsner, 1991	25°58′S 111°24′E	22
Kirsner et al., 1992	26°01′S 111°16′E 26°01′S 111°20′E	12
		15
Kirsner and Hughes, 1993	~26°13′S 111°25′E	17
**STAGE 2: COGNITION—CONVERGING OPERATIONS**		
Kirsner, 1997a	26°15′S 111°E	10
Kirsner and Dunn, 1998a	26°15′S 111°E	10
Finding Sydney Foundation, 2001	~26°06′S 110°52′E	11
Finding Sydney Foundation, 2003	~26°10′S 111°10′E	7
**STAGE 3: COGNITION—DECISION MODEL**		
Kirsner and Dunn, 2004, 2008; Dunn and Kirsner, 2011; King, 2014; Kirsner and Dunn, 2014	26°04′S 111°02′E	3
Finding Sydney Foundation, 2005: Acknowledged Kirsner and Dunn	26°04′S 111°02′E	3
Finding Sydney Foundation, 2007: Acknowledged Kirsner and Dunn	26°04′S 111°02′E	3

**Figure 9:**
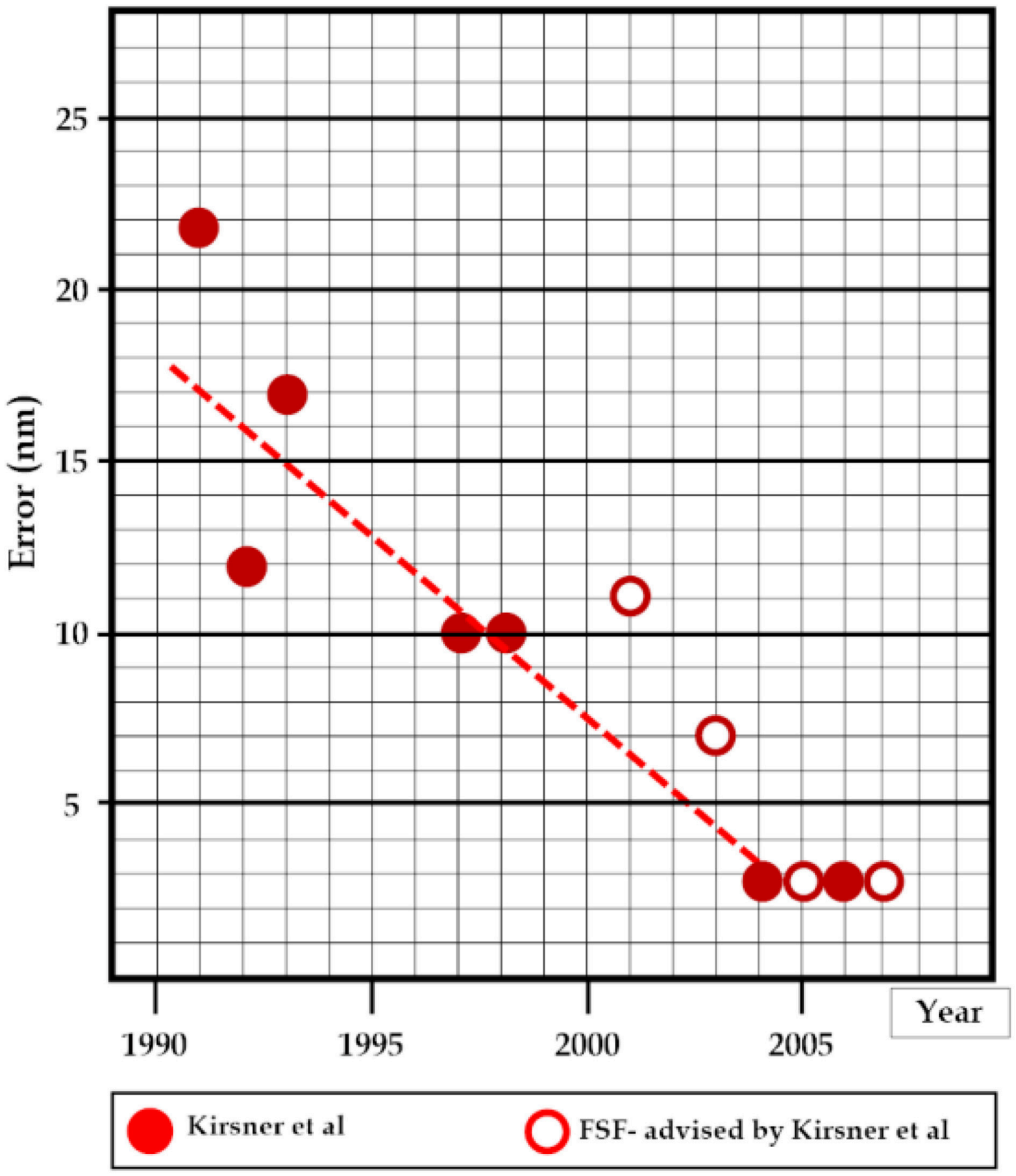
**Performance (Error) for the author and his colleagues between 1991 and 2005**.

The foregoing analysis described the collection and analysis of evidence concerning the location of the wreck of *Kormoran*. The improvement in performance summarized in Table 2 and Figure 9 does not reflect the performance of either a single individual or a regular team in the traditional sense of these terms. The task of wreck-hunting lies somewhere on a continuum of decomposability. At one extreme, the slow, and fundamental changes involved in landing safety on aircraft carriers (Wiegmann and Shappell, 2003, p. 5) and construction time for Liberty ships during World War II (Searle and Gody, 1945). Involved massively decomposable tasks where dozens or even hundreds of people contributed to the improvement in performance. The skills associated with accurate kicking for an oval-shaped Australian Football League football can be decomposed for learning purposes but they cannot be distributed across players or experts during a game. Each one has to kick the ball for himself or, on rare occasions, herself.

Figure 1 identified a number of discipline-specific approaches to wreck-hunting, several of which were adopted by the author and his colleagues. The learning curve observed in Figure 9 arguably reflects transitions across domains, from oceanography (1991–1993) to history to the cognitive sciences including adoption of a formal decision model. The critical drivers reflected: first, the construction of a substantial database; second, decisions about the viability of the report data; and, third, adoption of informal and then formal approaches to the use of multiple constraints. The research also reflected a coherent and evolving approach to a clearly defined problem concerning the location of the wreck of *Kormoran*, and, critically, it reflected input from a variety of disciplines, domains and scientists, individuals with diverse backgrounds.

Figure 9 includes the positions that the author and his colleagues tabled between 1991 and 2004. All of the positions in the plot are shown against an ordinate that indicates the distance between the position recommended and the position of the wreck of *Kormoran*, and the plot therefore reflects learning or skill acquisition. The reports formed three obvious groups involving oceanography, informal analysis of the Kormoran Database, and formal or mathematical instantiation of the Minimum Distance Principle on the database respectively. The model tested a potentially infinite range of candidate locations, and selected the position that involved the smallest possible amount of movement for the set of nine constraints. The one and only solution associated with the third stage of the project therefore involved 26°04′S 111°02′E, a position just 2.7 nm from the wreck of *Kormoran*.

## Expertise

The path of improvement from 1991 to 2005 reflected input from no fewer than three disciplines, oceanography, history, and the cognitive sciences. One implication of this perspective is that the research involved both a *horizontal* trajectory, as we accepted and understood the limitations and opportunities associated with oceanographic and historical research respectively, and a *vertical* trajectory, as we implemented successive more and more powerful cognitive analyses of the survivors' reports. In so far as the project involved a *vertical* skill acquisition path, it conformed to the tradition established by John Anderson more than 30 years ago (e.g., Anderson, 1982), as well as the more specific benefits associated with transfer involving component process models, models that might or might not cross domain boundaries (e.g., Speelman and Kirsner, 2005).

The *horizontal* trajectory reflects an argument advanced by Engeström and his colleagues (e.g., Engeström and Sannino, 2010). According to Engeström (2014) for example,

“*Learning by Expanding* challenges traditional theories that consider learning a process of acquisition and reorganization of cognitive structures within the closed boundaries of specific tasks or problems.”

Elsewhere, Engeström (1996) proposed that learning is not restricted to “vertical movement across levels” but should also be viewed as “horizontal movement across borders.” From a cognitive perspective however, the boundaries between the domains and the skills can be inherently fuzzy, and improvement will depend on comprehension and practice at the level of the component processes, and the discipline behind a given process might or might not be critical.

People acquire expertise or skill over a more or less unlimited range of domains and problems. The shear variety of the domains encompassed by human enterprise is formidable, and few attempts have been made to provide a *universal* model; that is, a model that covers all realms of human activity. To list but five disparate topics, a universal model would need to cater for the acquisition of skill or expertise in everything from cigar-rolling (Crossman, 1959) to survival in aerial combat (Spick, 1989), teamwork on the navigation bridge of a notional escort carrier (Hutchins, 1996), the reduction of flying accidents on Aircraft Carriers over 50 years (Wiegmann and Shappell, 2003) and construction times for Liberty ships (Searle and Gody, 1945). Wreck-hunting is just another cab off the ranks in the drive to describe and understand expertise and, if possible, define not only universal principles, such as the power law of learning, but a universal taxonomy as well.

**Figure 10:**
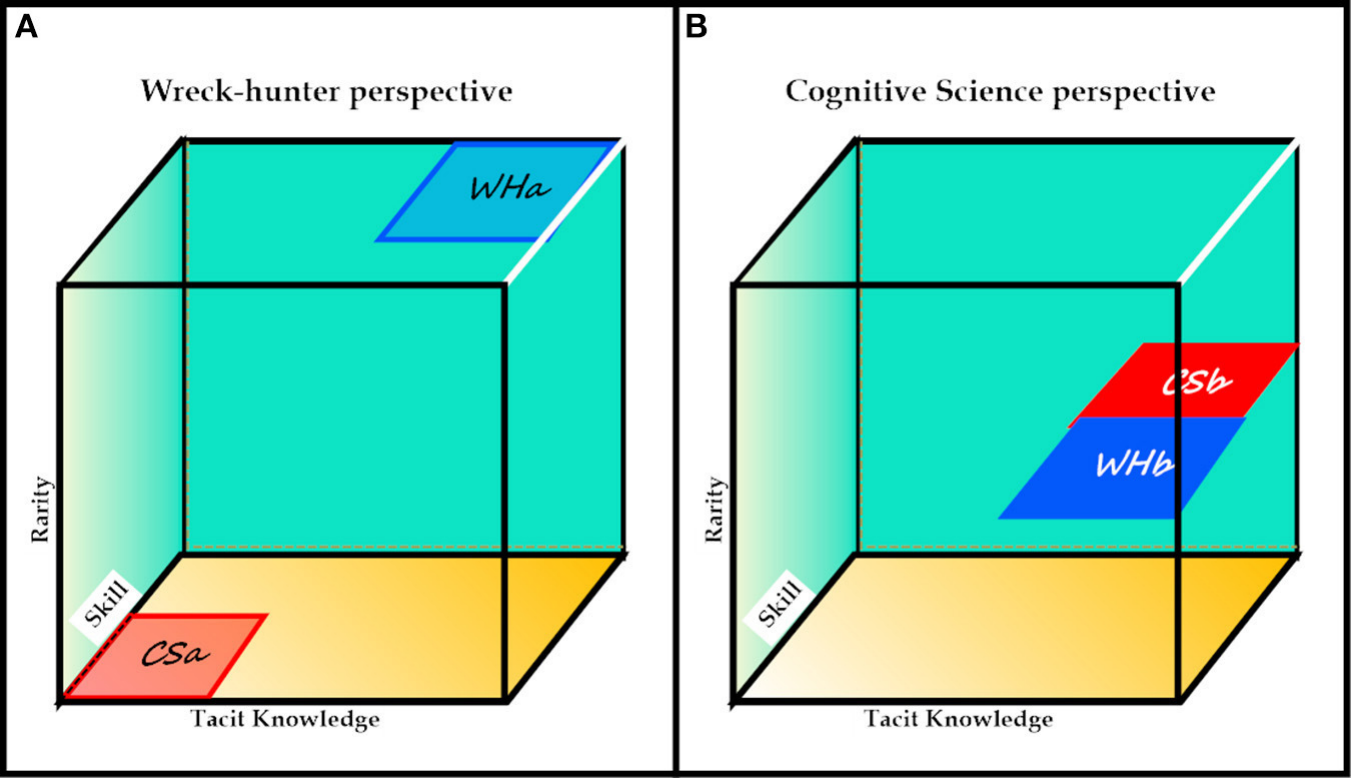
**Three dimensional model of expertise (from Collins, 2013)**. **(A)** reflects classification by a notional wreck-hunter. **(B)** reflects classification by the author, a classification that depends on the assumption that expertise for wreck-hunting is open to decomposition, a fourth dimension.

Collins (2013) has offered a useful starting point in regard to a universal taxonomy with a three-dimensional model of expertise. The model was introduced under the heading of *Studies of Expertise and Experience*, and Figure 10 honors the *Expertise-Space Diagram* depicted by that author.

The dimensions described by Collins were as follows:

1. The first or diagonal dimension is referred to as “Individual or group accomplishment” by Collins but the author has adopted a more traditional approach, treating this dimension as “Skill Acquisition,” or, more simply, Skill, a term that usually pre-supposes qualitative changes in information processing strategies or processes as individuals or groups transit from novice to expert.
2. The second dimension described by Collins refers to the “transmission of domain-specific tacit knowledge,” or Tacit Knowledge, involving either groups or individuals. The dimension is referred to as Tacit Knowledge in Figure 10, and depends on “immersion in the society of those who already possess it” (Collins, 2013, p. 3).
3. The third dimension referred to by Collins is “Esotericity,” and this dimension is depicted on the vertical axis in Figure 12. According to Collins (2013, p. 5).

“While traditional analyses take the word “expert” to refer only to rare, high-level, specialists, SEE (i.e., the model described by Collins) considers that ordinary language-speaking, literacy and the like exhibit a high degree of expertise even though everyone has them—they are ubiquitous. This is, perhaps, one of the most radical contributions of SEE to the analysis of expertise as indicated by the initial strong opposition to the idea of “ubiquitous expertise” from philosophers and psychologists. Part of the task of this paper will be to try to make it obvious that the idea of ubiquitous expertise is a necessity if we are to avoid confusion.”

In the following analysis, and in Figure 10, the term *rarity* is preferred to *esotericity* because of its frequency in natural language and its quantitative roots. A critical issue raised by Collins concerned the *rarity* of the relevant skills or expertise in his three-dimensional model of expertise. Collins questioned the traditional view that experts are of necessity “unusual individuals who have self-consciously devoted many hours of their lives to gaining a special ability.” Instead, and based in part on the proposition that all native speakers of a language are experts to a greater or lesser extent in their native language, Collins mounted an argument against the esoteric or rarity characteristic of expertise, and proceeded to assert that “the idea of ubiquitous expertise is a necessity.” Later, when faced with the challenge posed by racing car drivers, he proposed that the relevant skills form a body distinct from that of driving in general.

The importance of *decomposition* is evident. Changes in construction times for Liberty ships built in the US ranged from about 1.2 million man-hours per ship in the early days to less than 0.5 million man-hours per ship after 2000 or more vessels had been constructed. While a team of experts would have been essential to the design, co-ordination and management of each project, the improvement in ship-building times reflected many and widely distributed forms of expertise.

Another type of skill that reflects practice involved the performance and survival of fighter pilots in World Wars I and II (See Spick, 1989). Spick, for example, depicted the extent to which the probability of survival as a fighter pilot increased as a function of missions completed. Task analysis in this case involved a totally different picture from ship-building. The task of flying and fighting in World War I aircraft depended on indivisible expertise, expertise that accumulated with combat experience. The role of decomposition is quite different in this case however. While decomposition would have been possible and even desirable for instruction and training purposes, it was not possible to spread the skill across individuals under combat conditions, and each individual fighter pilot had to bring a full suite of skills to bear on the combat problem. Thus, while expertise can be distributed across thousands of engineers and craftsmen for ship-building, and reflect skill acquisition for the corporate entities as well as the individual tradesmen, a very different story applied to the performance of fighter pilots during World Wars I and II, and decomposition was not feasible under operational conditions.

But where does the foregoing analysis leave wreck-hunting in regard to expertise, or indeed, any research challenge that involves or could involve trans-disciplinary forms of expertise? The owners of the traditional forms of expertise might be reluctant to include provision for trans-disciplinary expertise, particularly if their background did not prepare them for challenges of this type.

Figure 10A depicts the model that a professional wreck-hunter might assert, and the model that was asserted or endorsed by virtually all of the parties involved the search for *Kormoran* and *Sydney*. However, as implied in the foregoing analysis, wreck-hunting can be treated as a decomposable example of expertise, involving a series of semi-independent skills or components. The critical issue implied by Figure 10B is that the set of reports from the Kormoran Database was open to analysis and interpretation by any one of a large number of cognitive scientists. In many cases we would have required support from historians and linguists but that can be assumed for multi- or trans-disciplinary projects. Figure 10 therefore provides two frames of reference for a discussion of expertise in wreck-hunting; that of the wreck-hunter who claims that he or she is the only person who can solve the problem, and that of the cognitive scientist who claims that wreck-hunting can be decomposed into component skills, skills that are widely distributed in the scientific community.

The argument outlined in this section has significant ramifications for the agencies and individuals responsible for *unprecedented* challenges such as those faced by the officials associated with the searches for *Kormoran* and *Sydney*, and, more recently, Malasia Airlines 370. The decision space should not be dominated by mate-ship and political expediency. Where inter- and trans-disciplinary opportunities are or might be relevant, effective leadership should involve scientifically informed and flexible leadership.

## Converging operations, trading zones, and “enactive” cognition

The author is not aware of any past attempts to consider or review wreck-hunting as a domain of expertise. The challenge is further complicated by the fact that it depends on several more specific forms of expertise, and few people will enjoy the complete set of skills involved. The project outlined in this paper therefore involved a de facto “trading zone” (See Thagard, 2005), or, to be more specific, an attempt to exchange ideas and approaches among navigators, oceanographers, historians, and cognitive scientists. The solution actually involved an expansion of triangulation, with nine as distinct from three Lines or Estimates of Position. However, the general principles guiding the cognitive approach to the challenge remained stable throughout the research, and relied on the presence of multiple constraints to negate the uncertainty and possible error associated with many if not all of the available reports. Given the central role of triangulation, a task that traditionally involved the use of maps, rulers, and Lines of Position, our analysis provides an interesting fit to the framework offered by Enactive Cognition (Froese et al., 2012). Specifically, it involves a task where the “cognitive agents” implement triangulation to solve a problem—to define the location of a wreck—and the physical vehicle for implementation, be it in a map, a head or a computer, is of secondary importance. Thus, triangulation constituted the critical scaffold for prediction, and the deep challenge facing us as scientists involved the selection and, if necessary, refinement of new Lines or Position. The solution was also consistent with earlier lines of argument involving: publications and papers describing the search and rescue solution, the SAR/Oceanography solution published by Sam Hughes, the Sunda Strait to Fremantle tracks taken by *Sydney* on earlier voyages, and the lifeboat tracks from the probable point of disembarkation from Kormoran to the coast.

As argued by Thagard (2005), science has changed out of recognition over the course of the twentieth century. Whereas, the early days of the century witnessed the establishment of the now traditional disciplines and divisions, some of which have been retained in the current curricula of universities, many of the critical advances in science and technology reflected migration to the boundaries of the established disciplines, as, like memes, they embarked on new inter-disciplinary journeys of their own. These transformations are particularly clear in the new and rapidly changing sciences, and the industries behind them, underwater target detection and forensic science being two obvious examples. It is also very clear in medical science and in medical training, where the nature and application of knowledge are undergoing similar transformations.

Much of the work described in this article reflected the author's origins in the cognitive sciences but it also capitalized on concepts, practices, and assumptions from older disciplines, involving oceanography and history in particular. Furthermore, and as argued by Thagard, trading zones are likely to flourish when they involve “people, places, organizations, ideas, and methods,” and the arcane world of wreck-hunting provided a fascinating and challenging “trading zone.”

## Conclusion

The critical issue discussed in this article concerned the location of the wreck of the German raider *Kormoran* off the coast of Western Australia. An accurate solution required cognitive analysis of a chaotic database comprising more than 70 reports, a decision preceded by decisions to set aside oceanographic, navigation, map dowsing, historical and oral history arguments. The procedure reflected exceptional collaboration involving three or possibly more “trading zones,” a critical step for innovation in science.

### Conflict of interest statement

The author declares that the research was conducted in the absence of any commercial or financial relationships that could be construed as a potential conflict of interest.
